# Liberation From Mechanical Ventilation in the Cardiac Intensive Care Unit

**DOI:** 10.1016/j.jacadv.2022.100173

**Published:** 2023-01-27

**Authors:** Andi Shahu, Soumya Banna, Willard Applefeld, Penelope Rampersad, Carlos L. Alviar, Tariq Ali, Adriana Luk, Elaine Fajardo, Sean van Diepen, P. Elliott Miller

**Affiliations:** a Section of Cardiovascular Medicine, Yale School of Medicine, New Haven, Connecticut, USA; b Department of Medicine, Yale School of Medicine, New Haven, Connecticut, USA; c Division of Cardiology, Duke University School of Medicine, Durham, North Carolina, USA; d The Tomsich Family Department of Cardiovascular Medicine, Cleveland Clinic, Cleveland, Connecticut, USA; e The Leon H. Charney Division of Cardiovascular Medicine, New York University Langone Medicine Center, New York, New York, USA; f Division of Pulmonary and Critical Care, Mayo, Rochester, Minnesota, USA; g Peter Munk Cardiac Centre, Toronto General Hospital, University Health Network, Toronto, Ontario, Canada; h Division of Pulmonary, Critical Care, and Sleep Medicine, Yale University School of Medicine, New Haven, Connecticut, USA; i Department of Critical Care and Division of Cardiology, Department of Medicine, University of Alberta, Edmonton, Alberta, Canada

**Keywords:** cardiac intensive care, mechanical ventilation

## Abstract

The prevalence of respiratory failure is increasing in the contemporary cardiac intensive care unit (CICU) and is associated with a significant increase in morbidity and mortality. For patients that survive their initial respiratory decompensation, liberation from invasive mechanical ventilation (IMV) and the decision to extubate requires careful clinical assessment and planning. Therefore, it is essential for the CICU clinician to know how to assess and manage the various stages of IMV liberation, including ventilator weaning, evaluation of extubation readiness, and provide post-extubation care. In this review, we provide a comprehensive approach to liberation from IMV in the CICU, including cardiopulmonary interactions relative to withdrawal from positive pressure ventilation, evaluation of readiness for and assessment of spontaneous breathing trials, sedation management to optimize extubation, strategies for patients at a high risk for extubation failure, and tracheostomy in the cardiovascular patient.

## Highlights

• Respiratory insufficiency remains the primary cause for admission to the modern CICU.
• Liberation from IMV in the CICU requires careful planning and evaluation.
• Research incorporating the unique CICU patient population is needed to better define optimal treatment paradigms.

Respiratory failure in the contemporary cardiac intensive care unit (CICU) is becoming increasingly common.^1^^,^^2^ Upward of one-third of all admissions to the CICU now require some form of respiratory support.^3^ While it can be a lifesaving intervention, invasive mechanical ventilation (IMV) also carries several risks, including ventilator-associated injury (both microtrauma and macrotrauma), infections, and ICU-acquired weakness from immobilization.^4^^,^^5^ For patients that survive their initial respiratory decompensation, liberation from IMV is a potentially perilous time in a patient’s clinical trajectory. Extubation failure, including in patients passing a spontaneous breathing trial (SBT defined) (Table 1), is independently associated with a significant increase in mortality, estimated between 25 and 50%.^6^ Therefore, the CICU clinician must balance the potential complications of prolonged IMV with the risks of extubation failure and subsequent reintubation. In this review, we detail the cardiopulmonary interactions of positive pressure ventilation (PPV) and its withdrawal, as well as the key time periods for liberation from IMV (Central Illustration).

**Table 1:** Important Terminology Associated With Liberation From Mechanical Ventilation

Term	Definition
Spontaneous breathing trial	Assessment a patient’s readiness for extubation by evaluating ability to breathe with reduced or no ventilator support
T-piece	Type of SBT in which patient is disconnected from the ventilator with only oxygen supplementation
Pressure support	Type of SBT in which patient is placed on ventilator setting that provides additional inspiratory and/or expiratory support
Continuous positive airway pressure	Type of SBT in which patient is placed on ventilator setting that provides a continuous level of PEEP
Ventilator weaning	The process of progressive withdrawal of ventilator support
PEEP	Pressure above atmospheric pressure that is present in the lungs at the end of the expiratory phase

PEEP = positive end-expiratory pressure; SBT = spontaneous breathing trial.

**Central Illustration:**
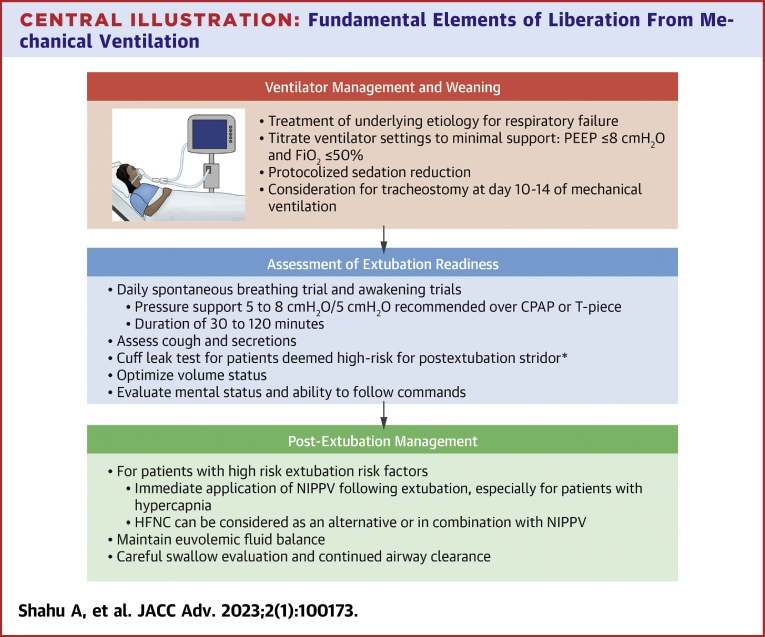
**Fundamental Elements of Liberation From MechanicalVentilation** ∗Traumatic intubation, intubation > 6 days, large ETT tube, female, and reintubation after unplanned extubation. CPAP = continuous positive airway pressure; FiO_2_ = inspired fraction of oxygen; HFNC = high flow nasal cannula; NIPPV = noninvasive positive pressure ventilation; PEEP = positive end-expiratory pressure.

## Spontaneous awakening and breathing trials

Appropriateness for liberation from IMV in the CICU can be assessed using an SBT, which should be coupled with a spontaneous awakening trial (SAT).^7^ The SAT refers specifically to the interruption of sedatives, ideally performed daily to help reduce oversedation, delirium, and help facilitate eventual liberation from IMV. Prior studies have shown that daily SATs are safe and do not lead to patient harm.^7^^,^^8^ Patients randomized to a paired SAT and SBT in the ABC (Awakening and Breathing Controlled) trial had fewer ventilator days, lower length of stay, and lower mortality compared to sedation per usual care and a daily SBT.^7^ While SATs can be performed without a concurrent SBT to help minimize use and possible harms associated with sedation, SBTs should be performed in conjunction with SATs to allow the patient the best chance of passing the SBT. A subsequent trial found that a daily SAT compared to protocolized light sedation did not reduce the duration of IMV or ICU length of stay.^9^ While there is limited evidence for one specific sedation protocol over another, the American College of Chest Physicians/American Thoracic Society (Chest/ATS) Clinical Practice Guidelines recommend protocolized light sedation for patients ventilated for >24 hours.^10^

The concept of SBT readiness testing was born out of the need for a structured approach to identify when patients may be ready for liberation from IMV. The application of objective criteria has been shown to reduce the duration of IMV and ICU length of stay.^11^ In 2001, a taskforce defined the fundamental elements necessary for “discontinuation potential” or readiness for an SBT.^12^ These simple criteria, detailed in Table 2 with additional cardiovascular considerations, identify hemodynamically stable and spontaneously breathing patients on minimal ventilator support, with improvement in their underlying etiology of respiratory failure.^12^

**Table 2:** Spontaneous Breathing Trial Readiness Criteria and Additional Cardiovascular Considerations

Original Criteria	Additional CV Considerations
Improvement or resolution of original cause of respiratory failure	Control of both atrial (rate and/or rhythm) and ventricular arrhythmias
Sufficient oxygenation on minimal ventilator settings^a^	Treatment of or stability of critical coronary disease
pH ≥7.25	Reasonable and improving left ventricular filling pressures
Hemodynamic stability^b^	Evaluation of insertion site and need for mechanical circulatory support
Ability of the patient to initiate their own breath	

CV = cardiovascular; PEEP = positive end-expiratory pressure.

a Typically defined as a PEEP ≤5 to 8 cmH_2_0 and fraction of inspired oxgyen ≤40% to 50%.

b No or low dose vasopressors (defined as dopamine or dobutamine <5 μg/kg/min in the original publication,^12^ but consideration for all vasopressors should be incorporated).

While these criteria have been validated in subsequent studies, CICU patient populations are poorly represented.^7^^,^^13^ Therefore, applying these criteria also warrant consideration of a patient’s cardiovascular hemodynamics, including volume status. For example, while early practice suggested continuing vasopressor use precluded weaning trials, subsequent studies have shown no deleterious effect on extubation success with ongoing vasopressors.^14^^,^^15^ In addition, SBTs can be physiologically demanding,^16^ and optimizing volume status and blood pressure may be of particular importance in the CICU patient population. Lastly, when assessing readiness for ventilator liberation in patients with recent atrial or ventricular arrhythmias, consideration must be made to ensure quiescence is achieved through adequate electrolyte replacement and antiarrhythmic management prior to initiating an SBT, which can cause recurrence from the increased physiologic stress of the trial itself.

During an SBT, the ventilator is changed from a mode that provides full ventilatory support (eg, volume-assist control or pressure-assist control) to a mode that provides a decreased amount or even no respiratory support.^17^ If the patient exhibits signs of respiratory failure during the SBT (Table 3), then they are deemed not ready for extubation. Conversely, a patient who passes their SBT without signs of respiratory distress may be ready for extubation. Though numerous studies have been conducted to compare different SBT approaches and modalities, many of the results are mixed, and practices vary regarding both the mode and duration of SBTs.^18^ Finally, there are several markers used to predict how likely an extubation is to succeed, which may inform the decision of whether to extubate the patient.

**Table 3:** Common Parameters Used to Identify Spontaneous Breathing Trial Failure

Vital Signs	Clinical Signs
HR >140 beats/min or >20% increase from baseline	Worsening mental status (both agitation and/or somnolence)
RR <6 or >35 breaths/min	Diaphoresis
SBP <90 or >180 mmHg	Increased work of breathing^b^
SaO_2_ <88%-90%^a^	Cyanosis
	Development of arrhythmias

FiO_2_ = inspired fraction of oxygen; HR = heart rate; RR = respiratory rate; SaO_2_ = oxygen saturation; SBP = systolic blood pressure.

a With FiO_2_ ≥0.50.

b Accessory muscle use, thoracoabdominal paradox.

**Figure 1:**
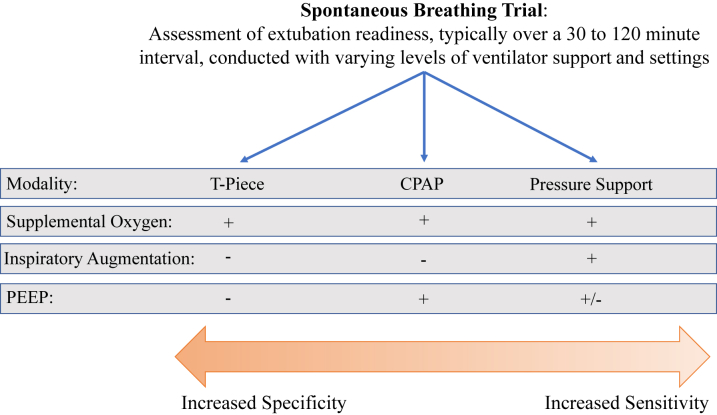
**Common Modalities Used During Spontaneous Breathing Trials** CPAP = continuous positive airway pressure; PEEP = positive end-expiratory pressure.

Over the last 30 years, several studies, often with mixed results, have tried to identify the most effective SBT modality. Historically, there have been 3 common modalities used for SBTs (Figure 1). During a T-piece test, the endotracheal tube is disconnected from the ventilator, and an adapter provides flow-by supplemental oxygen. Continuous positive airway pressure (CPAP) trials deliver only positive end-expiratory pressure (PEEP). Finally, pressure support trials provide inspiratory augmentation with or without PEEP. A meta-analysis suggested that T-piece or CPAP are more representative of post-extubation physiology and could better predict extubation success.^16^ However, others have found that the likelihood or rates of successful extubation are not significantly different regardless of whether a pressure support or T-piece strategy is employed, though patients who are placed on T-piece ventilation may be more likely to fail SBT.^19^ Other reports have suggested that passing an SBT with pressure support is a better predictor of a successful extubation than a T-piece trial.^20^^,^^21^ In addition to the chosen modality, the duration of the SBT, typically conducted over 30 to 120 minutes, is important. Compared to 2 hours, a 30-minute SBT has been found to be equally effective at predicting extubation success.^22^^,^^23^ Recently, a large (n = 1,153) randomized controlled trial (RCT) compared pressure support for 30 minutes versus a T-piece trial for 2 hours. They found that the shorter pressure support trial was associated with higher rates of extubation success (82.3% vs 74.0%, *P* = 0.001), as well as a lower hospital and 90-day mortality.^24^ Recent clinical practice guidelines recommend that patients who have been ventilated for at least 24 hours should receive an SBT with 5 to 8 cmH_2_O of inspiratory pressure support (conditional recommendation, moderate quality evidence).^25^ While these guidelines are largely based on data from patient populations that may not be generalizable to the CICU, we would recommend that most patients in the CICU undergo an SBT with low level pressure support and that 30 minutes should be sufficient in almost all cases (Central Illustration).

Once a modality is chosen and the SBT has begun, the CICU clinician should monitor for parameters of SBT failure as well as assess for potential predictors of extubation success. While no single parameter should be used in isolation; a combination of several objective and subjective criteria has commonly been used to identify SBT failure (Table 3).^26^ In addition to vital signs and clinical assessment, there are a variety of physiologic measures that have been used as predictors of successful extubation with the most cited factor being the rapid shallow breathing index (RSBI).^26^ First described in 1991, the RSBI was defined as the ratio of respiratory rate to tidal volume measured over 1 minute with spontaneous breaths using a T-piece. An extubation was considered more likely to be successful if the RSBI was <105 breaths/min/L.^27^ Although a sensitive test (83%), the specificity is poor (58%),^28^ and while useful, it should not be the sole predictor used to evaluate extubation success/candidacy. In fact, when used as the primary decision tool, it has been shown to prolong intubation without reducing mortality or risk of extubation failure.^13^ There are numerous other physiologic measures, but their poor predictive value often limits their clinical usefulness and are not recommended by the current guidelines.^10^ In addition, there are numerous multicomponent indices (eg, CORE index, integrative weaning index, etc),^29^^,^^30^ which can be accurate predictors of extubation success but are cumbersome to measure.

Failure of an SBT should prompt a comprehensive evaluation of possible underlying etiologies of SBT failure (Table 4). A patient may not pass an SBT due to abnormal respiratory mechanics (eg, increased resistive load from bronchospasm), diaphragmatic deconditioning or atrophy, delirium or oversedation, and/or underlying cardiovascular dysfunction.^17^ As soon as a patient has been deemed to fail their SBT, they should immediately be put back on a “non-fatiguing” ventilator mode, such as assist-control, as diaphragmatic fatigue can take upward of 24 hours to recover.^31^ While conducting a second SBT later in the day is not unreasonable if the cause of failure is clear, this approach has not been found to improve extubation success.^32^ Accordingly, we would recommend once daily SBTs, unless there is an easily reversible etiology for failure of the first SBT (eg, oversedation during the first test, uncontrolled arrhythmia, etc).

**Table 4:** Reasons for SBT Failure and Investigations to Address the Underlying Etiology

Reason for SBT Failure	Assessment and Treatment
Weaning induced pulmonary edema	• Assess volume status (physical exam, chest x-ray, bedside ultrasound, PAC) • Maintain negative fluids balance with intravenous diuretics
Oversedation	• Protocolized assessment and titration of sedation • Stop continuous infusions of and/or consider intermittent opioid regimens • Discontinue benzodiazepines
Delirium	• Non-pharmacologic management for delirium reduction (eg, reorientation, sleep hygiene, etc) • Pharmacologic therapy for agitated delirium
Bronchospasm	• Administer bronchodilating medications • Assess response with changes in peak pressures
Unresolved pulmonary infection	• Confirm pneumonia via imaging or sputum cultures • Bedside ultrasound to assess for pleural effusions • Treatment with antibiotics and ensure adequate clearance of secretions
Uncontrolled arrhythmia	• Control (rate and/or rhythm) of atrial arryhthmias • Ensure any ventricular arrhythmias are quiescent before repeating SBT
Diaphragmatic deconditioning	• Consider diaphragmatic ultrasound • Wait 24 h for next SBT • Consider higher support settings for next SBT

PAC = pulmonary artery catheter; SBT = spontaneous breathing trial.

## Utility of bedside ultrasound

The use of bedside ultrasonography, both lung and cardiac imaging, has become increasingly commonplace in critical care settings to help assess for SBT and/or extubation failure. In a meta-analysis of echocardiography parameters, diastolic dysfunction and elevated left ventricular filling pressures have been found to be associated with SBT failure. Specifically, elevated peak early mitral inflow velocity (E) to early diastolic mitral annular velocity (e’), higher E waves, and lower e’ waves were associated with SBT failure.^33^ At least one study previously reported on the utility of stress echocardiography (using either dobutamine or ephedrine) to unmask diastolic dysfunction or significant mitral regurgitation. In patients that failed their SBT, dobutamine administration was associated with an increase in E/e’ as well as a decrease in strain and strain rate assessed by speckle tracking (all, *P* = 0.001). Similarly, ephedrine use was able to reproduce the increase in afterload from the SBT and unmask worsening mitral regurgitation.^34^ However, it is important to note that many of the studies assessing echocardiogram parameters and extubation failure were generally small, non-randomized, conducted in non-CICU units, and excluded or poorly represent patients with systolic dysfunction. Our institution is currently conducting a prospective trial (EXTUBATE-ECHO) of echocardiogram measurements in patients with an ejection fraction ≤40% to predict SBT failure, which should provide more information for typical CICU patients.

Extra-cardiac imaging, including lung and diaphragmatic ultrasound, has also been used to predict successful liberation from IMV. The lung ultrasound (LUS) score and modified LUS are 2 validated predictors of extubation success. The LUS includes 12 regions scored from 1 to 4, and the modified LUS score uses 8 regions scored 0 to 5 with higher values in both scores representing an increased risk of extubation failure.^35^ Diaphragmatic excursion (DE) and diaphragmatic thickening fraction (DTF) are the 2 primary measures of diaphragm function assessed by ultrasound. DE is the distance the diaphragm moves during an unassisted breath, and DTF is a ratio of diaphragmatic thickness in inspiration and expiration.^35^ In a meta-analysis of 19 studies, DTF was found to be a more accurate predictor of weaning outcomes than DE, which is more modifiable by external factors like patient positioning and abdominal pressure.^36^

## Cardiopulmonary interactions with liberation from positive pressure ventilation

**Figure 2:**
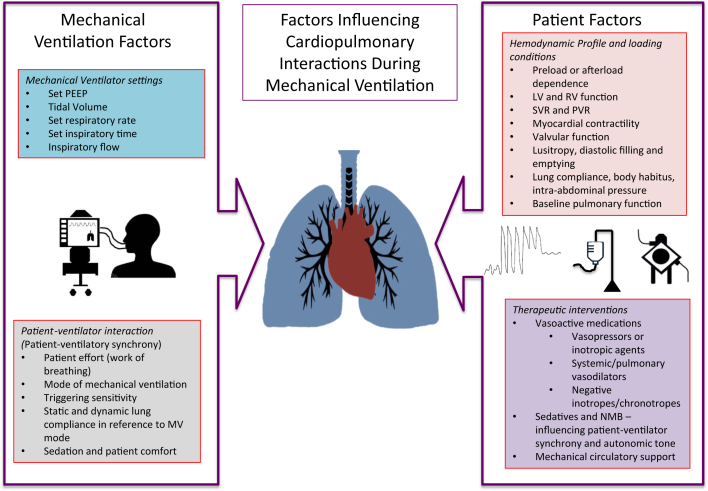
**Factors Influencing Cardiopulmonary Interactions During Mechanical Ventilation** LV = left ventricular; PEEP = positive end-expiratory pressure; PVR = pulmonary vascular resistance; RV = right ventricular; SVR = systemic vascular resistance.

The effects of PPV on the cardiovascular system are determined by several factors (Figure 2).^37^ These include parameters related to the mechanical ventilator itself, which can influence intrathoracic pressures, and in turn alter preload and afterload. Second, the mode of mechanical ventilation, level of support, specific mechanical properties of the patient’s respiratory system, and patient-ventilator synchrony influence the extent of hemodynamic consequences and respiratory effort. Third, the patient’s individual hemodynamic and loading conditions, including preload and afterload, myocardial contractility, factors influencing stroke volume such as valvular function or left ventricular outflow tract (LVOT) obstruction, and determinants of myocardial diastolic filling such as pericardial disease or restrictive physiology may affect a patient’s response PPV.^38^^,^^39^ Fourth, pharmacological interventions affecting hemodynamics, including vasoactive medications, sedatives, neuromuscular blockers, and mechanical circulatory support may also influence the hemodynamic response to PPV.

**Figure 3:**
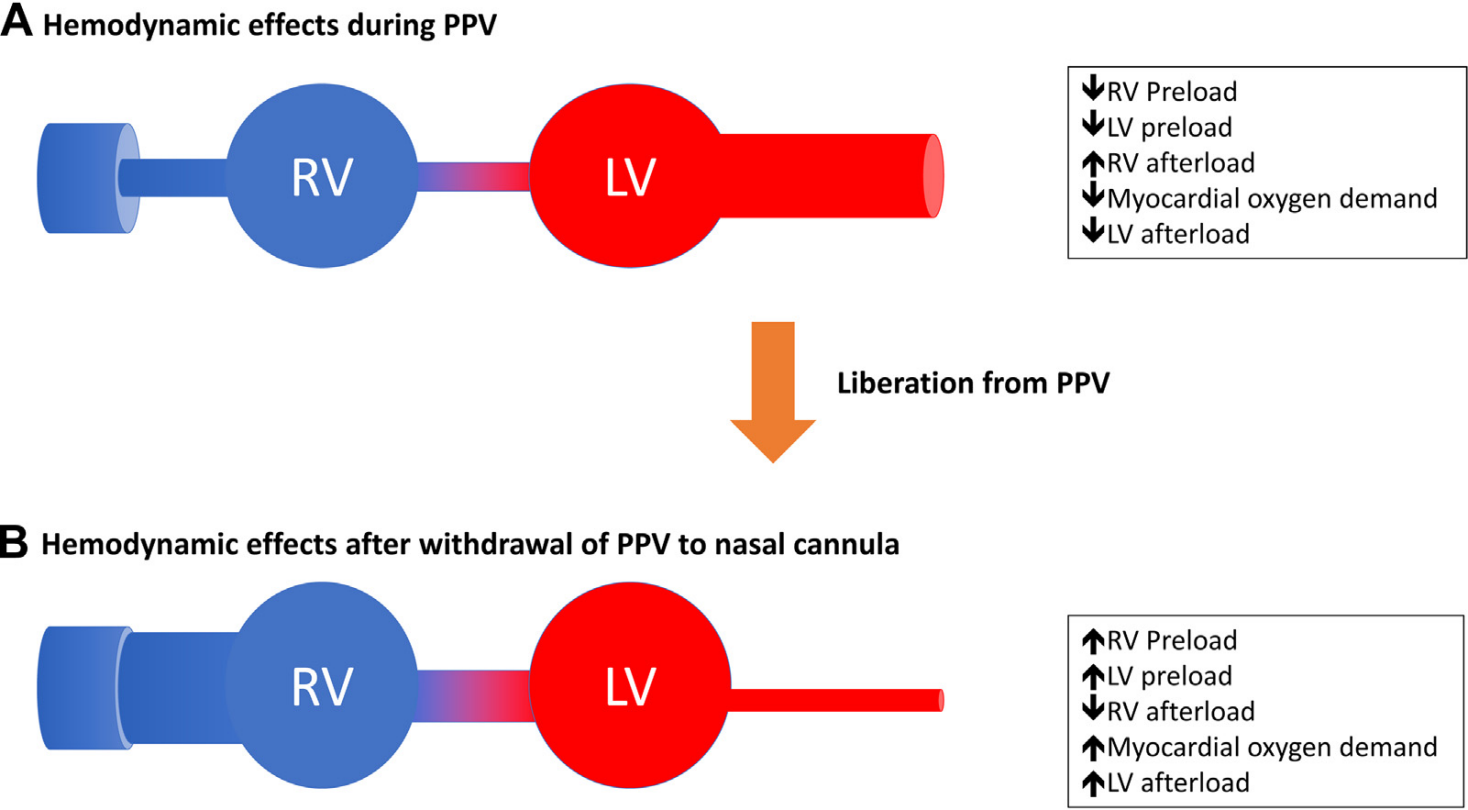
Hemodynamic Effects of Positive Pressure Ventilation on the Cardiovascular System Hemodynamic effects on the cardiovascular system with **(A)** positive pressure ventilation (PPV) and **(B)** withdrawal of PPV. **(A)** PPV decreases the pressure gradient between the capacitance vessels and the right atrium resulting in a reduction in preload, or venous return, to both the right ventricle and subsequently the left ventricle (LV). PPV favorably improves LV energetics by decreasing LV transmural tension, myocardial oxygen demand, and afterload. **(B)** Withdrawal of PPV can lead to an increase in both RV and LV preload as well as a reduction in RV afterload. In comparison, LV afterload and subsequently myocardial oxygen demand may increase.

The main factor that influences cardiopulmonary interactions during the transition from IMV to spontaneous breathing is the removal of PPV (the basics of which have been well described,^37^^,^^40^ and detailed in Figure 3). During SBTs, cardiopulmonary interactions are primarily determined by the set level of support provided, which also ultimately influences patient-ventilator synchrony. Compared to a T-piece SBT, which provides the least amount of support, the addition of PEEP and escalating levels of pressure support is associated with a >30% to 40% reduction in patient effort.^41^^,^^42^ Alternatively, due to the increase in preload and afterload from the cessation of PPV, SBTs without PEEP and inspiratory augmentation are potentially associated with an increase in PCWP and the development of pulmonary edema.

Several unique scenarios are worth further explanation. In patients with a dynamic LVOT obstruction, SBTs without inspiratory pressure support or PEEP might actually improve LVOT gradients and improve hemodynamics, as “negative pressure ventilation” from unsupported breaths will increase LV afterload and reduce dynamic outflow obstruction.^39^ Similarly, PEEP titration through reductions in right ventricular afterload may also be beneficial for preload-dependent states (eg, right ventricular failure, tamponade, restrictive physiology, hypertrophic obstructive cardiomyopathy). In contrast, PPV may be favorable to the patient with reduced ejection fraction, aortic insufficiency, and mitral regurgitation by reducing LV afterload and encouraging forward flow. However, breathing trials and liberation from IMV may actually unmask mitral regurgitation and lead to extubation or SBT failure.^43^ These dynamic changes related to liberation from IMV highlight the importance of understanding each patient’s individual physiology and to adjusting the IMV liberation process accordingly.

Following extubation, withdrawal of PPV can lead to an abrupt increase in LV afterload and preload (Figure 3B), increasing the risk of acute pulmonary edema, especially for patients with ischemic heart disease or ventricular dysfunction.^38^^,^^44^ This can potentially be offset by the immediate transition to noninvasive PPV (NIPPV),^42^ particularly using NIPPV to match the last setting used in the pressure support mode and targeting both clinical stability and patient comfort. Similarly, hemodynamic tailoring, including optimizing preload with diuretics or venodilators (eg, nitrates), afterload reduction with arteriolar vasodilators (eg, sodium nitroprusside, hydralazine, renin-angiotensin-aldosterone system inhibitors) and myocardial contractility (eg, inotropic agents) can also allow for a smoother transition and successful liberation from IMV. Despite benefit in select patients, NIPPV is contradicted or should be used with caution in patients with altered mental status unable to protect their airway, facial deformities/trauma preventing an adequate seal, recent facial or upper airway surgery, hemodynamic instability, and patients prone to aspiration with active emesis or inability to clear secretions.^37^

## Difficult to liberate patients

Despite efforts to optimize patients’ hemodynamics and respiratory physiology prior to extubation, between 19% and 25% of patients will experience delayed liberation from IMV.^45^ There are multiple approaches to liberate the difficult to wean patient, and there is wide variation in practice patterns across ICU types, centers, and countries.^46^ The first, and most common, would be to investigate for reversible causes of SBT failure, further optimize a patient’s physiology, and then attempt an SBT the next day.^31^

For the smaller proportion of patients with prolonged IMV, there are several potential strategies distinct from repeating daily SBTs. In a landmark trial, Brochard et al randomized patients who failed a 2-hour SBT to a strategy of synchronized intermittent mandatory ventilation (SIMV), pressure support, or progressive T-piece weaning. Compared to SIMV and T-piece, pressure support was found to have a lower number of weaning failures (23% pressure support, 42% SIMV, 43% T-piece, *P* = 0.05). A second trial which also compared the 3 previously mentioned modalities found that the rate of successful weaning was higher with those randomized to T-piece SBTs compared to SIMV or pressure support weaning protocols.^32^ These discordant results likely reflect substantial differences in the study protocols, and while these are the best available evidence for difficult to liberate patients, we note that many of these trials were conducted more than 2 decades ago, when daily SBT trials were not the standard of care. As a result, the generalizability of these strategies to the CICU population is not entirely certain.

Newer techniques for weaning include advanced closed loop ventilation modes, such as proportional assist ventilation (PAV) and neurally adjusted ventilatory assist (NAVA). Both of which are proprietary and not available on all ventilator brands. PAV uses the Equation of Motion (P_airway_ − P_musc_ = Flow · Resistance + Volume/Compliance + PEEP) to estimate work of breathing during each breath.^47^ Clinicians dictate the percent work of breathing per breath that they wish the ventilator to perform. In NAVA, an esophageal probe measures the electrical activity of the diagram, which allows the clinician to titrate the level of pressure the ventilator delivers per patient effort.^47^ These advanced ventilatory modes have been compared favorably to more traditional pressure support trials and can be helpful in determining if the patient is ready for extubation.^48^ In a systematic review of studies comparing closed loop ventilation modes to the traditional pressure support, utilization of NAVA was associated with lower in-hospital and ICU mortality as well as a lower likelihood of NIPPV use in the first 48 hours after extubation. Meanwhile, PAV utilization was associated with a higher likelihood of successful liberation from the ventilator, shorter IMV duration, and ICU length of stay.^48^

Regardless of the modality chosen, it is critical that a readiness assessment and SBT, if the patient is eligible, be conducted daily. For the CICU patient that fails their SBT, they should immediately be placed on a non-fatiguing ventilator mode (typically set to volume/pressure-control or pressure support, as per European guidelines),^31^ which minimizes work of breathing and ventilator dyssynchrony.

## Considerations after a successful SBT

**Figure 4:**
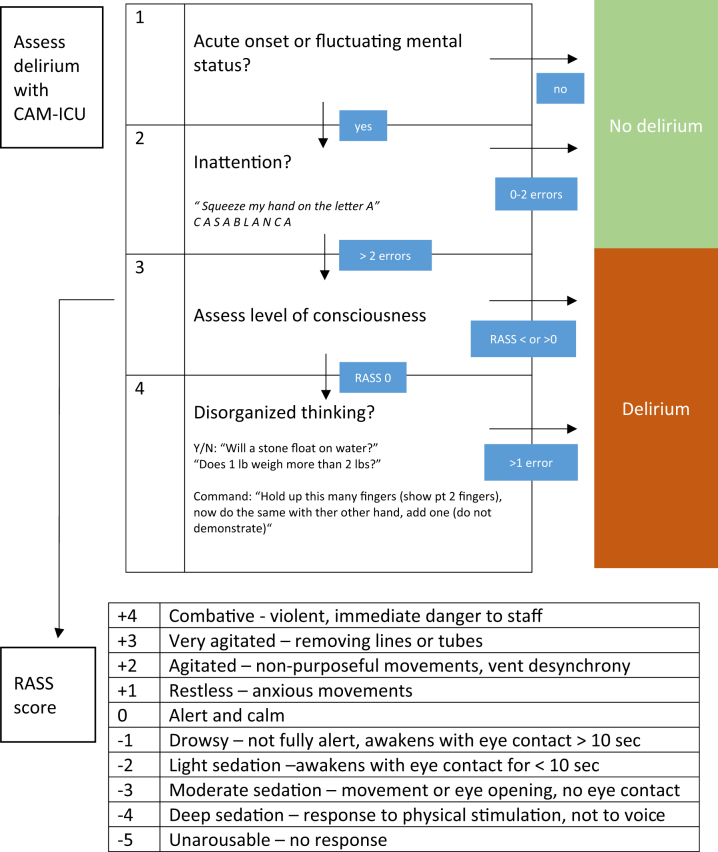
**Integration of the CAM-ICU and Glasgow Coma Scale** CAM = Confusion Assessment Method; ICU = intensive care unit; RASS = Richmond Agitation-Sedation-Scale.

Reduced level of consciousness is one of several clinical parameters that can be used to assess readiness for extubation following a successful SBT. Traditionally, the Glasgow Coma Scale (GCS) has been used, with a score ≥8 suggestive of a higher likelihood of success,^49^ though a more comprehensive assessment including the ability to follow commands (i.g., closing eyes, showing 2 fingers, wiggle toes, or coughing on command) together with a clinical assessment of delirium using the Confusion Assessment Method for the Intensive Care Unit (CAM-ICU) or Intensive Care Delirium Screening Checklist (ICDSC) score may also be used to assess neurological status (Figure 4).^50^^,^^51^ A meta-analysis in the neurocritical care unit showed that the inability to follow commands is associated with a higher rate of extubation failure.^52^ Although a GCS ≥8 has been found to be more successful than a GCS <7 in neurosurgical patients (75% vs 37%, *P* < 0.0001), this population is substantially different than the CICU patient population. We would suggest that patients should ideally be extubated with a GCS ≥9-T (intubated) with either no or well controlled delirium.

Presence of a cough and gag reflex is important to assess before extubation. The presence of a cough reflex suggests adequate muscle tone of the diaphragm and intercostal muscles and suggests the patient will likely be able to clear their own secretions. Higher rates of reintubation have been found in patients with high volume of respiratory secretions. In one study, copious secretions at the time of extubation were an independent predictor of extubation failure.^53^ Though it remains difficult to quantify secretion volume, one study found that patients with secretions exceeding 2.5 mL/h were 3 times more likely to fail liberation compared to those with less secretions.^54^ Peak cough flow, as measured by external spirometry or by a flow meter within the ventilator, has been used to predict success of extubation, with values >160 L/min being favorable to avoid extubation failure.^55^ A meta-analysis analyzing 12 studies has shown that a cough peak flow of <55 to 65 L/min is a strong predictor of extubation failure (normal: 360-400 L/min). Thus, given that copious respiratory secretions could portend an increased risk of respiratory failure, the presence of an effective cough may serve as a mitigating factor against extubation failure. Additionally, a pharyngeal gag reflex demonstrates a patient’s ability to protect their airway and its absence may suggest significant brain injury.^56^

Laryngeal edema can occur as early as 36 hours into endotracheal intubation. When present, it can be associated with post-extubation stridor and can increase the likelihood of reintubation.^57^ As a provider cannot assess the upper airway easily, an endotracheal cuff leak, detecting the presence of audible expired air around the endotracheal tube can be done qualitatively at the bedside. A quantitative assessment can also be done by measuring the expiratory tidal volume loss on the ventilatory while the endotracheal cuff is deflated. A recent meta-analysis, however, found a sensitivity and specificity of a cuff leak test for post-extubation airway obstruction of 0.62 and 0.87, respectively, suggesting that those with a detectable cuff leak still need to be monitored post-extubation for airway obstruction.^58^

When laryngeal edema is suspected, corticosteroids can be given pre-extubation. There is no standard corticosteroid regimen as data comparing corticosteroid regimens are limited. However, compared to placebo, RCTs have shown a reduction in reintubations with various corticosteroid regimens, which have included methylprednisolone 40 mg intravenous (IV) 4 hours before extubation, 4 doses of 40 mg IV methylprednisolone every 6 hours over 24 hours, or dexamethasone 5 mg IV every 6 hours over 24 hours.^59^, ^60^, ^61^ Chest/ATS guidelines recommend systemic steroids at least 4 hours before extubation for patients with a failed cuff leak.^62^ To minimize fluid retention in those at risk (eg, patients with heart failure), one dose of methylprednisolone could be considered.

In summary, once the CICU patient has passed an SBT and volume status and hemodynamics have been optimized, the CICU clinician should assess for delirium, secretions, cough, and a cuff leak.^37^ Neurologically, patients should ideally have a GCS ≥9-T with no or well controlled delirium, minimal secretion burden with adequate cough strength, and the presence of a cuff leak. If the adjunctive assessment described above following an SBT confirms a high rate of liberation success, the CICU clinician should evaluate if a difficult airway was documented at the time of intubation. If so, the intubation technique should be reviewed, and necessary equipment and trained personnel should be available if needed post-extubation.

## Sedation management near extubation

The management of pain, anxiety, and agitation in intubated patients is critical but can delay liberation from IMV if not carefully managed. Sedation strategies should balance minimizing patient discomfort while avoiding oversedation. Common sedatives include benzodiazepines, propofol, and dexmedetomidine (Table 5).^63^^,^^64^ Benzodiazepines may convey some hemodynamic advantages, particularly in patients with cardiogenic shock, as they do not depress cardiac output and may even reduce cardiac filling pressures.^65^ However, studies have shown that benzodiazepines are an independent risk factor for delirium in ICU patients,^66^ and may lead to prolonged IMV and longer ICU length of stay compared to other agents.^67^^,^^68^ Thus, utilization of benzodiazepines should be limited in the CICU, except for special circumstances, such as those experiencing seizures or alcohol withdrawal.^69^ The Society of Critical Care Medicine guidelines recommend utilization of non-benzodiazepine agents, including propofol or dexmedetomidine, as the preferred choice in ICU patients.^63^ Meanwhile, opioids are first-line analgesic agents for intubated patients and should be transitioned to as needed intermittent dosing, if not done already, as the patient approaches extubation readiness.

**Table 5:** Sedation Medications Used During the Peri-Extubation Period

Drug Class	Drug Name	Typical Drug Dosing	Adverse Effects
General anesthetic	Propofol	Bolus: 0.25-2.0 mg/kg IV Infusion: 5-50 μg/kg/min IV	Hypotension, bradycardia, negative inotropy, propofol infusion syndrome (rare), hypertriglyceridemia; caution if LVEF <50%; cannot perform SBT while on propofol
Alpha-2 adrenergic agonist	Dexmedetomidine	Loading: 1 μg/kg over 10 min IV Infusion: 0.2-1.5 μg/kg/h IV	Hypotension, bradycardia; caution if patient has existing heart block or bradycardia; can perform SBT while on dexmedetomidine
Analgesic agents/Opioids	Fentanyl	Loading: 25-100 μg Maintenance intermittent dosing: 25-50 μg q 30-60 min Infusion: 25-200 μg/h IV	Hypotension, bradycardia, fentanyl-induced chest wall rigidity (rare), hypertension, tachycardia; caution morphine use in preload dependent states (eg, RV failure) due to venodilatory effect; QTc prolongation (methadone)
	Hydromorphone	Bolus: 0.2-1 mg q1-3 h IV Infusion: 0.5-4.0 mg/h IV	
	Morphine	Bolus: 2-10 mg IV Infusion: 2-30 mg/h IV	
	Methadone	2.5-10.0 mg q8-12 h IV or 10-40 mg q6-12 h PO	
Anxiolytic agents	Midazolam	Bolus: 0.01-0.05 mg/kg IV Infusion: 0.01-0.10 mg/kg/h IV	Delirium, hypotension, propylene glycol toxicity (lorazepam)
	Lorazepam	Bolus: 0.02-0.04 mg/kg (max dose 4 mg) IV Infusion: 0.01-0.10 mg/kg/h	
Antipsychotic agents	Quetiapine	50-200 mg BID PO	QTc prolongation, hypotension, hypertension, tachycardia
	Haloperidol	Bolus: 0.5-20.0 mg IV or IM Infusion: 0.5-2.0 mg/h IV	

Adapted from Alviar et al,^37^ Barr et al,^63^ and Tietze et al.^3^

BID = twice per day; IM = intramuscular; IV = intravenous; LVEF = left ventricular ejection fraction; PO = per os (by mouth); RV = right ventricle; SBT = spontaneous breathing trial.

Propofol is a frequently utilized agent for sedation in the CICU. It has been shown to result in less delirium and fewer days of IMV when compared with benzodiazepines.^70^^,^^71^ Propofol has a favorable pharmacokinetic profile with rapid onset and can be quickly weaned off as its plasma concentration rapidly declines when the infusion is held, enabling frequent awakening trials.^71^ Propofol may also aid in terminating supraventricular and ventricular tachycardias through sympathetic inhibition. As such, propofol may be preferred for deep sedation in patients with incessant ventricular tachycardia.^72^ However, due to its sympathetic inhibition, propofol may have dose-dependent hemodynamic effects, resulting in hypotension through negative inotropy, chronotropy, and vasodilatation.^69^^,^^73^ Other side effects include hypertriglyceridemia (triglycerides must be monitored regularly) and propofol infusion syndrome (a rare but possibly lethal complication).^74^ Propofol utilization has not been exclusively studied in the CICU, though it has been studied in patients following cardiac surgery and is generally considered to be a safe and effective sedative in patients who have cardiac comorbidities.^75^ However, we encourage careful titration of propofol in patients with cardiogenic shock or significant ventricular dysfunction.

Dexmedetomidine, an alpha2-adrenergic receptor agonist, is an alternative agent which reduces the risk of delirium as well as time to extubation when compared with both benzodiazepines and propofol.^67^^,^^75^ Dexmedetomidine is an excellent anxiolytic, particularly, when hyperactive delirium is considered prohibitive to proceeding with an SBT.^76^ However, adverse effects, such as hypotension, bradycardia, and drug fever may be higher compared to other agents.^67^ The overall safety profile of dexmedetomidine use does not appear to be significantly different for CICU vs medical ICU populations.^77^ One single-center CICU study found approximately 60% of patients on dexmedetomidine experienced hypotension.^77^ However, this study was small and did not compare adverse events of other sedatives. Dexmedetomidine should be avoided in patients with high-degree heart block and used with caution in those with bradycardia (HR <50 beats/min).

Utilization of systematic assessments of sedation and delirium has been shown to significantly reduce the duration of IMV.^78^ The RASS (Richmond Agitation-Sedation-Scale) is a widely accepted tool used to assess the level of sedation in intubated patients (Figure 4). Patients should also be screened regularly for delirium using a validated screening tool, such as CAM-ICU or ICDSC.^78^, ^79^, ^80^ The incidence of delirium in the CICU has ranged from 8 to 20%, depending on definitions and assessment tools.^81^^,^^82^ Like other ICUs, delirium is associated with significant morbidity and mortality.^78^ However, the CICU has several specific challenges, including a higher proportion of patients with acute decompensated HF and cardiac arrest who are particularly predisposed to delirium,^83^ along with frequent use of immobilizing therapies, such as mechanical circulatory support and temporary transvenous pacemakers.^84^ Delirium precautions and investigation for reversible causes of delirium are considered first line, and if antipsychotic agents are used, there should be close monitoring of the QTc.

Several unblinded RCTs have compared outcomes among patients randomized to standardized sedation protocols versus sedation without a protocol.^10^ Although results from these trials vary in the overall effectiveness of sedation protocols, the use of a sedation protocol carries a conditional recommendation (low quality of evidence) from the Chest/ATS guidelines. Regardless of the institutional sedation protocol used, a daily SAT, often paired with an SBT, has been found to reduce the duration of IMV, ICU length of stay, and potentially mortality.^7^^,^^8^ A recent RCT including 700 patients compared “nonsedation” (bolus morphine allowed for analgesia) to light sedation (RASS −2 to −3) with daily sedation interruptions. The mean RASS score in the nonsedation arm was approximately one, depending on the day of ventilation. They found no difference in ICU-free days, ventilator-free days, or 90-day mortality.^85^ Although the applicability to the CICU patient population, especially those with cardiogenic shock or cardiac arrest, is unclear, this study does highlight the importance of lighter sedation targets, especially when nearing extubation, and the potential use of opioid only sedation.

In summary, sedation management for mechanically ventilated patients in the CICU should incorporate frequent use of multidisciplinary sedation protocols, delirium management, and transition from continuous to intermittent dosing when possible. While there is no optimal first-line sedative agent in the CICU, the choice of sedative should be individualized to a patient’s underlying cardiovascular disease and hemodynamic profile. As the patient nears liberation from IMV, preference should be given for either dexmedetomidine or propofol.^86^ Since it does not decrease respiratory drive, dexmedetomidine can be continued after extubation, whereas medications like propofol must be stopped. Regardless of the chosen sedative, daily awakening with interruption of sedation remains the standard of care in intubated patients.

## Extubation strategies

Once a CICU patient has passed an SBT and is deemed ready for extubation, consideration should be given for extubating to “prophylactic” respiratory support, such as NIPPV or high-flow nasal cannula (HFNC). The goal of respiratory support following liberation from IMV is to prevent extubation failure and reintubation. In a multicenter CICU registry, the incidence of reintubation was 7.6% at a median of 2 days.^3^ While extubation failure is likely a marker of overall acuity, some evidence suggests that extubation failure is an independent risk factor for mortality, with mortality rates upward of 50%.^6^^,^^87^, ^88^, ^89^, ^90^ Resource limitations often dictate that NIPPV or HFNC cannot be used in all post-extubation patients, and the use of these devices should be reserved for the population where benefit has been shown. Patients at high risk for respiratory failure post-extubation are elderly, with underlying cardiac disease, respiratory disease or hypercapnia (Table 6).

**Table 6:** Potential Risk Factors for Extubation Failure in Patients Who Have Passed a Spontaneous Breathing Trial

Cardiac Risk Factors	Noncardiac Risk Factors
Heart failure (especially with LVEF ≤30%)^91^	Prolonged IMV (>7 d)^91^
Cardiac etiology for respiratory failure^6^	Chronic lung disease^89^^,^^92^
Positive fluid balance in the preceding 24 h^93^	Weak cough^91^^,^^94^^,^^95^
BNP increase <20% during SBT^96^	Age >65 y^89^
Reduction in central venous saturation >4.5% during SBT^97^	Abundant secretions^a^^,^^94^^,^^98^
	Glasgow Coma Scale ≤10^98^
	Hemoglobin ≤10 g/dL^94^
	Lack of cuff leak^57^

BNP = brain natriuretic peptide; IMV = invasive mechanical ventilation; LVEF = left ventricular ejection fraction; SBT = spontaneous breathing trial.

a Required frequent suctioning (eg, every 1-2 hours) or >2.5 mL/h.

The time to initiation of these devices post-extubation is key, as the use of NIPPV or HFNC after the development of respiratory distress has shown little benefit and likely harm.^99^^,^^100^ In comparison, there is considerable evidence that immediately applying respiratory support to high-risk patients after extubation can reduce reintubation.^101^, ^102^, ^103^ Two RCTs compared NIPPV to oxygen therapy in patients at high risk for reintubation. Both found that prophylactic NIPPV was associated with a lower risk of reintubation and ICU mortality.^101^^,^^102^ Given a subgroup analysis which showed the improved outcomes may be partly driven by hypercapnic patients (defined as an arterial PaCO_2_ >45 mmHg), a second trial of patients that had chronic respiratory disease and hypercapnia after a successful SBT demonstrated that prophylactic NIPPV was associated with a reduction in reintubation and 90-day mortality.^103^ For high-risk patients, 2017 joint European and American guidelines give a conditional recommendation for prophylactic NIPPV (moderate certainty of evidence).^104^

More recently, prophylactic HFNC has become an alternative to reduce the risk of reintubation.^105^ HFNC provides a constant flow of heated and humidified oxygen at up to 1.0 of inspired fraction of oxygen and flows up to 70 L/min. There are several physiologic benefits of HFNC, including reduced anatomical dead space, improved work of breathing, and potentially low levels of PEEP (2-5 cmH_2_O) at higher flows. However, it is important to note that HFNC is an open system, and levels of PEEP are lower and perhaps negligible when the patient’s mouth is open.^106^^,^^107^ In a study that randomized 105 patients with a partial pressure of oxygen/inspired fraction of oxygen ratio <300 mmHg to a Venturi facemask or HFNC for 48 hours post-extubation, patients randomized to HFNC reported better interface comfort, reduced oxygen desaturations, and decreased reintubations.^108^ HFNC has also been compared to NIPPV post-extubation in high-risk patients. Neither the rate of reintubation nor the median time to reintubation was significantly different between those randomized to HFNC or NIPPV. Of note, 100% of patients randomized to HFNC tolerated the intervention for 24 hours, compared to a median tolerated time of 14 hours for patients randomized to NIPPV.^109^ However, the applicability of this study to the CICU population is unclear, given heart failure was the primary indication for IMV in only 7.8% of their cohort. In addition, only one-third of patients had any heart disease.^109^ More recently, Thille et al compared the combination of HFNC with NIPPV with HFNC alone in high-risk patients. In the combination group, NIPPV was applied immediately after extubation for at least 4 hours with HFNC during breaks. Reintubation was significantly less frequent in the combination group than in the HFNC only group.^110^ Still, while 47% of the study population had at least one underlying cardiovascular comorbidity, only 14% and 7% of patients had left ventricular dysfunction or a history of cardiogenic pulmonary edema, respectively.

Current cardiovascular societal guidelines lack guidance on extubation strategies. However, a recent American Heart Association Scientific Statement recommended the use of NIPPV for patients at risk of reintubation.^86^ The 2017 Chest/ATS Practice Guidelines recommend extubation to preventative NIPPV for high-risk patients receiving IMV for >24 hours (strong recommendation, moderate grade of evidence).^10^ Neither gives a recommendation for HFNC. Finally, the European Society of Intensive Care Medicine recommends HFNC over conventional oxygen therapy but recommends the continued use of NIPPV for patients typically deemed high-risk (conditional recommendation, low certainty evidence).^105^ Taken together, the predominance of evidence and expert recommendations lean toward NIPPV for patients deemed high-risk of reintubation due to cardiovascular dysfunction. As detailed above, currently available RCTs may not accurately represent the patient population in the CICU. In practice, HFNC offers an attractive option for patients with contraindications or intolerance to a full facemask or where there is a concern for potential emesis.

In addition to consideration for extubation to respiratory support, the CICU clinician must also consider both the timing of extubation after a successful SBT as well as CICU specific logistical issues, such as the frequent placement of arterial femoral lines for mechanical circulatory support or hemodynamic monitoring.^83^ Time of day (day vs night) can also be very important. In a retrospective study of approximately 100,000 patients including 165 ICUs, night extubations were associated with more frequent reintubations (14.6% vs 12.4%, *P* < 0.001) and higher in-hospital mortality (16.0% vs 11.1%, *P* < 0.001), which persisted after a propensity-matched analysis.^111^ When considering extubation at night, the staffing model in the CICU, etiology of respiratory failure, and length of IMV should be considered.

The presence of mechanical circulatory support, including with a femoral insertion site, is not a contraindication to extubation. When IMV is deemed no longer necessary, CICU providers should assess for the continued need of femoral devices as an inability to sit upright may put the extubated patient at a mechanical disadvantage. However, if mechanical circulatory support is still required, every effort should be made to extubate or liberate the patient from IMV if they are ready. While mobility remains limited for patients with femoral placed percutaneous ventricular assist devices or intra-aortic balloon pumps, several single center and at least one multicenter study have reported on the safety and feasibility of extubations on extracorporeal membrane oxygenation (ECMO). Extubation while on ECMO may allow for improved mobilization, reductions in sedatives, and favorable outcomes,^112^ but requires a multidisciplinary team and careful planning.

## Tracheostomy

Upward of one-third of patients intubated for >48 hours in the medical ICU eventually receive a tracheostomy.^113^ While rates of tracheostomy in the heterogenous CICU patient population are unknown, one study which included nearly 200,000 patients with acute myocardial infarction complicated by cardiogenic shock found the incidence of tracheostomy to be 5.7%, including 15% of patients who were intubated for >96 hours.^114^ There are several potential benefits of tracheostomy, including reduced patient discomfort, reduced need for sedation, increased early mobilization, and improved weaning from IMV.^115^^,^^116^ However, the impact of tracheostomy on mortality and the ideal timing are still controversial.^113^ Furthermore, there is wide variation in timing based on patient populations (eg, trauma vs pneumonia) as well as center-specific patterns.^117^

Two RCTs may provide guidance on tracheostomy timing. One study randomized 419 patients receiving IMV for >48 hours to early (6-8 days) or late (13-15 days) tracheostomy. Only 69% of the patients in the early and 57% of the subjects in the late group underwent tracheostomy. Early tracheostomy was not associated with a statistically significant reduction in ventilator-associated pneumonia, ventilator days, ICU days, or mortality.^118^ Another, multicenter study randomized patients on IMV for <4 days and deemed likely to require at least 7 more days of IMV to early (within 4 days) compared to late (after 10 days) tracheostomy. They did not find a difference in mortality, ICU length of stay, or adverse events. In addition, clinician assessment of extended ventilator requirement was limited as only 44.9% of patients in the late group eventually required a tracheostomy.^119^ Taken together, we generally recommend waiting between 10 and 14 days before pursuing a tracheostomy, given mixed data and the limited clinical ability to predict prolonged ventilator requirements.

Of particular importance to the CICU population is tracheostomy management in patients on antiplatelets and anticoagulation. Depending on center expertise, potential options for tracheostomy include percutaneous and surgical approaches. The percutaneous approach has increased in popularity given that it can be done at the bedside with potentially lower costs and fewer complications.^120^ In a 2-center retrospective study, 20 patients who had received clopidogrel (16 on aspirin) at least 3 days prior to undergoing percutaneous tracheostomy had a similar risk of minor bleeding compared to 137 patients not on clopidogrel.^121^ Similarly, in another small study of 34 CICU patients, only minor bleeding was reported in 3 patients undergoing percutaneous tracheostomy.^122^ In another study of patients receiving systemic heparin (held for 1 hour before percutaneous tracheostomy) for ECMO, there were similar rates of bleeding and complications compared to other critically ill patients.^123^ Although the available data are limited to small, retrospective studies, bedside percutaneous tracheostomy can be considered in experienced hands on antiplatelets and anticoagulants if they absolutely cannot be held (eg, recent stent placement).

## Conclusions and future perspectives

Given the increasing prevalence of respiratory failure among patients in the CICU, it has become necessary for the CICU clinician to be well versed in ventilator physiology, ventilator-associated complications, as well as weaning and liberation from IMV. Liberation from IMV requires a multidisciplinary team approach, including coordination and input from all members of the clinical team. The CICU clinician should feel comfortable assessing for extubation readiness, optimizing hemodynamics in preparation for extubation, overseeing weaning from the ventilator, interpreting the results of SBTs, managing peri-extubation sedation, and identifying which patients may benefit from post-extubation respiratory support. Due to the unclear generalizability of non-CICU clinical trials and the unique challenges in the CICU patient population, future studies embedded in the CICU or with a higher representation of patients with cardiovascular disease are needed. In particular, studies evaluating the best ICU practices in the CICU setting, including the ideal SBT modality for patients with right and/or left ventricular dysfunction, further identification of markers of extubation success, sedation management around extubation, and identification of which patients benefit most from NIPPV or HFNC following extubation, are sorely needed.

## Funding support and author disclosures

Dr Miller has received research funding from Fisher & Paykel. All other authors have reported that they have no relationships relevant to the contents of this paper to disclose.
